# A Review: Gaseous Interventions for *Listeria monocytogenes* Control in Fresh Apple Cold Storage

**DOI:** 10.3389/fmicb.2021.782934

**Published:** 2021-12-09

**Authors:** Jiewen Guan, Alison Lacombe, Bhargavi Rane, Juming Tang, Shyam Sablani, Vivian C. H. Wu

**Affiliations:** ^1^Produce Safety and Microbiology Research Unit, Western Regional Research Center, Agricultural Research Service, United States Department of Agriculture, Albany, CA, United States; ^2^Department of Biological Systems Engineering, Washington State University, Pullman, WA, United States

**Keywords:** *Listeria monocytogenes*, food safety, fresh apples, cold storage, gaseous interventions

## Abstract

*Listeria monocytogenes* (*L*. *monocytogenes*) causes an estimated 1600 foodborne illnesses and 260 deaths annually in the U.S. These outbreaks are a major concern for the apple industry since fresh produce cannot be treated with thermal technologies for pathogen control before human consumption. Recent caramel apple outbreaks indicate that the current non-thermal sanitizing protocol may not be sufficient for pathogen decontamination. Federal regulations provide guidance to apple processors on sanitizer residue limits, organic production, and good manufacturing practices (GMPs). However, optimal methods to control *L*. *monocytogenes* on fresh apples still need to be determined. This review discusses *L*. *monocytogenes* outbreaks associated with caramel apples and the pathogen’s persistence in the environment. In addition, this review identifies and analyzes possible sources of contaminant for apples during cold storage and packing. Gaseous interventions are evaluated for their feasibility for *L*. *monocytogenes* decontamination on apples. For example, apple cold storage, which requires waterless interventions, may benefit from gaseous antimicrobials like chlorine dioxide (ClO_2_) and ozone (O_3_). In order to reduce the contamination risk during cold storage, significant research is still needed to develop effective methods to reduce microbial loads on fresh apples. This requires commercial-scale validation of gaseous interventions and intervention integration to the current existing apple cold storage. Additionally, the impact of the interventions on final apple quality should be taken into consideration. Therefore, this review intends to provide the apple industry suggestions to minimize the contamination risk of *L*. *monocytogenes* during cold storage and hence prevent outbreaks and reduce economic losses.

## Introduction

Apples are one of the most valuable fruit crops in the United States (U.S.). The apple industry brings 5 billion dollars of revenue to the economy annually (U.S. Apple Association [USApple], 2021). In 2014, caramel apples contaminated with *L. monocytogenes* were linked to a foodborne illness outbreak in which 35 people across 12 U.S. states contracted listeriosis, 7 (20%) of which died (U.S. Centers for Disease Control and Prevention [CDC], 2014). Apples are commonly consumed raw or minimally processed (Du et al., 2002). There is no “kill step” included in the fresh produce postharvest packing process to eliminate pathogenic bacteria. The current fresh apple industry relies heavily on postharvest washing to control foodborne pathogens. However, the most commonly used sanitizer, chlorine (hypochlorite), has been reported to react with organic matters to form carcinogenic compounds, raising health and environmental hazards (Artés et al., 2009).

During harvest, apples are picked by hand in the orchards and transported in bins to the packinghouse, where they are either cold-stored or washed, sized, sorted, and packed for the retail market. Unwashed apples are moved into refrigerated storage – either short-term conventional refrigeration or long-term controlled atmosphere (CA) (Pietrysiak et al., 2019). *L. monocytogenes* has the ability to grow at refrigeration temperatures and is persistent in a cold environment (Walker et al., 1990). The environmental testing of the caramel apple outbreak showed that the contamination was introduced on the apples at the firm’s packing facility. Since then, several voluntary recalls have been reported for potential *Listeria* contaminations on fresh apples (U.S. Food and Drug Administration [FDA], 2017a, 2019a). These incidents show the need to improve current food safety systems in the apple packing industry. In particular, there is a need for developing and validating effective interventions to reduce contamination.

Several European countries such as Germany, Belgium, Denmark, Switzerland, and the Netherlands have banned chlorine in commercial produce washing (Artés et al., 2009; Shen et al., 2016). The efficacy of chlorine disinfection is highly dependent on the pH of the solution (Connell, 1996; Sun et al., 2019). Various environmentally friendly alternative sanitizers like chlorine dioxide, peracetic acid (PAA), and ozone have been suggested to replace chlorine (Rodgers et al., 2004; Hua et al., 2019; Guan et al., 2021). However, there has been limited information on the feasibility of those and other potential interventions for the apple industry.

Food safety interventions that minimize contamination risks are of critical importance. Therefore, the objective of this review is to identify the safety gaps during apple packing process and analyze the potential application of gaseous interventions in apple cold storage. An integrated approach to the existing apple packing facility is urgently needed. Currently, the federal agencies, the fresh apple industry, and researchers have switched their focus to gaseous interventions that decontaminate *L. monocytogenes* on fresh apples. This review will consider the regulatory requirements on gaseous interventions as well as organic production and handling of apples that could contribute to future industrial application and benefit the apple processors.

## Listeria Monocytogenes

### *Listeria monocytogenes* Outbreaks

Listeriosis is a serious infection caused by the consumption of *L. monocytogenes* contaminated food. U.S. Centers for Disease Control and Prevention [CDC] (2021) reports that an estimated 1,600 people have been diagnosed with listeriosis yearly, and the death rate is about 16% in the U.S. *Listeria monocytogenes* is a gram-positive, facultative anaerobic pathogenic bacterium. It has the ability to replicate at refrigeration temperatures. These characteristics help *L. monocytogenes* adapt to produce-associated environments (e.g., cold storage) where other bacteria might be prohibited to grow (Du et al., 2002). For example, in the caramel apple outbreak in 2014, all fresh Granny Smith and Gala apples were voluntarily recalled because environmental testing revealed contamination with *L. monocytogenes* at the firm’s apple-packing facility in California (U.S. Centers for Disease Control and Prevention [CDC], 2014). In January 2015, the U.S. Food and Drug Administration (FDA) conducted a traceback investigation. Six of the seven environmental samples positive for *L. monocytogenes* indistinguishable from the outbreak strains were isolated from food contact surfaces (FCS), including polishing brush, drying brushes, conveyor, and inside a wooden bin.

FDA investigation found the cross-contamination between FCS and apples likely played a role in the consecutive apple contamination in the outbreak. Thus, it has been hypothesized that *L. monocytogenes* contamination could happen throughout the packing process and the distribution chain. Once the pathogen is introduced to the environment, it is difficult to eliminate if appropriate good manufacturing practices (GMPs) are not applied. The *Listeria*-contaminated fresh produce caused foodborne illness and increased the food and economic loss for the produce industry, heightening awareness of food safety and implementing the Food Safety Modernization Act (FSMA). Fresh fruit growers, packers, and processors are required to adopt validated and effective preventive controls by the Produce Rule of the FSMA (U.S. Food and Drug Administration [FDA], 2011).

### Persistence in Environment

There are many opportunities for *L. monocytogenes* to attach to the produce surface in a typical apple packing house. In the investigation of the cantaloupe and caramel apple outbreaks, environmental contamination at packing houses and equipment FCS were likely the source of listeriosis outbreaks (U.S. Centers for Disease Control and Prevention [CDC], 2011, 2014). These include cross-contamination during washing, the wax coating unit operation, cold storage, and FCS like polishing brushes and dryer rollers (Ruiz-Llacsahuanga et al., 2021). Cooling and packing operations may also be responsible for bacterial contamination of the produce due to the significant amount of water utilized during the packing process. Wet surface areas in packing facilities are favorable for bacterial growth (Pietrysiak et al., 2019). Water may facilitate biofilm development as one of the main locations, which enhances the persistence of *L. monocytogenes* in the environment (Galié et al., 2018). *L. monocytogenes* may spread in the environment and lead to food contamination, which emphasizes the importance of good agriculture practices (GAPs), good manufacturing practices (GMPs), and HACCP for the produce postharvest handling and processing (Farber et al., 2003). Therefore, it is critical to identify the safety gaps during the packing lines and implement preventive interventions to avoid contamination.

*Listeria monocytogenes* has been isolated from soil, water, animal manure, and decaying vegetation (Farber and Peterkin, 1991; U.S. Food and Drug Administration [FDA], 2019b). Unlike other foodborne pathogens, *L. monocytogenes* can survive between a wide range of temperature (−0.4 to 50°C) and pH values (4.3 to 9.4) (Farber and Peterkin, 1991; Pietrysiak et al., 2019), and is resistant to adverse environmental conditions, such as low temperature, water activity or oxygen, and high acidity or salt (Buchanan and Phillips, 1990; Walker et al., 1990). The unique ability of *L. monocytogenes* to survive or even grow at low temperatures makes it a primary pathogen to contaminate fresh produce in refrigerated packing. *L. monocytogenes* persists in a produce packing environment from months to years, resulting in recontamination of the produce passing through that environment (Leong et al., 2017) and posing a high safety risk to the prolonged storage of fresh apples. The expression of cold shock proteins has been reported to help *L. monocytogenes* with the adaptation of low temperatures (Matereke and Okoh, 2020). Neunlist et al. (2005) concluded that the membrane lipids alteration of *L. monocytogenes* protects it from cold stress. In addition, whole-genome sequencing (WGS) showed that *Listeria* isolates from the voluntarily recalled whole apples, collected along the distribution chain, were highly related to the outbreak strains (U.S. Centers for Disease Control and Prevention [CDC], 2014; Angelo et al., 2017).

#### Biofilm Formation

Biofilms are complex structures composed of multiple cells embedded in an extracellular matrix that is mainly formed by polysaccharides, proteins, or extracellular DNA. This matrix can adhere to hard surfaces in a food processing environment, such as FCS (equipment, transport, storage surfaces, etc.) or food surfaces (vegetables, fruits, meat, etc.), responsible for the strong persistence of biofilm in the food industry. Biofilm formation offers the microbial cells higher physical resistance against desiccation, mechanical resistance against removal by liquid streams, and chemical resistance against antimicrobials and disinfectants (Galié et al., 2018). The presence of biofilm in food industry environments puts human health at risk. Thus, the food industry is seeking biofilm prevention and disruption methods.

*Listeria monocytogenes* biofilms are generally formed by teichoic acids. They can grow on major food contact surfaces, including stainless steel, low-density polyethylene (LDPE), polyvinyl chloride (PVC), polyester (PET), rubber, and glass surfaces throughout the food industry (Hua et al., 2019). Dygico et al. (2020) stated that factors like time, temperature, surface type, nutrient availability, and origin could affect the biofilm formation of *L. monocytogenes*. Then this pathogen can contaminate the food batches from the surfaces. With the replication ability at low temperatures, *L. monocytogenes* reinforces its hydrophilicity and induces biofilm formation as a response to cold temperatures, enhancing its attachment to surfaces and its resistance to sanitation procedures in food manufacturing plants. These characteristics highlight the great importance of inspecting and controlling *L. monocytogenes* biofilms in the food industry (Galié et al., 2018).

## Fresh Apple Packing Process

### After Harvest

**FIGURE 1:**
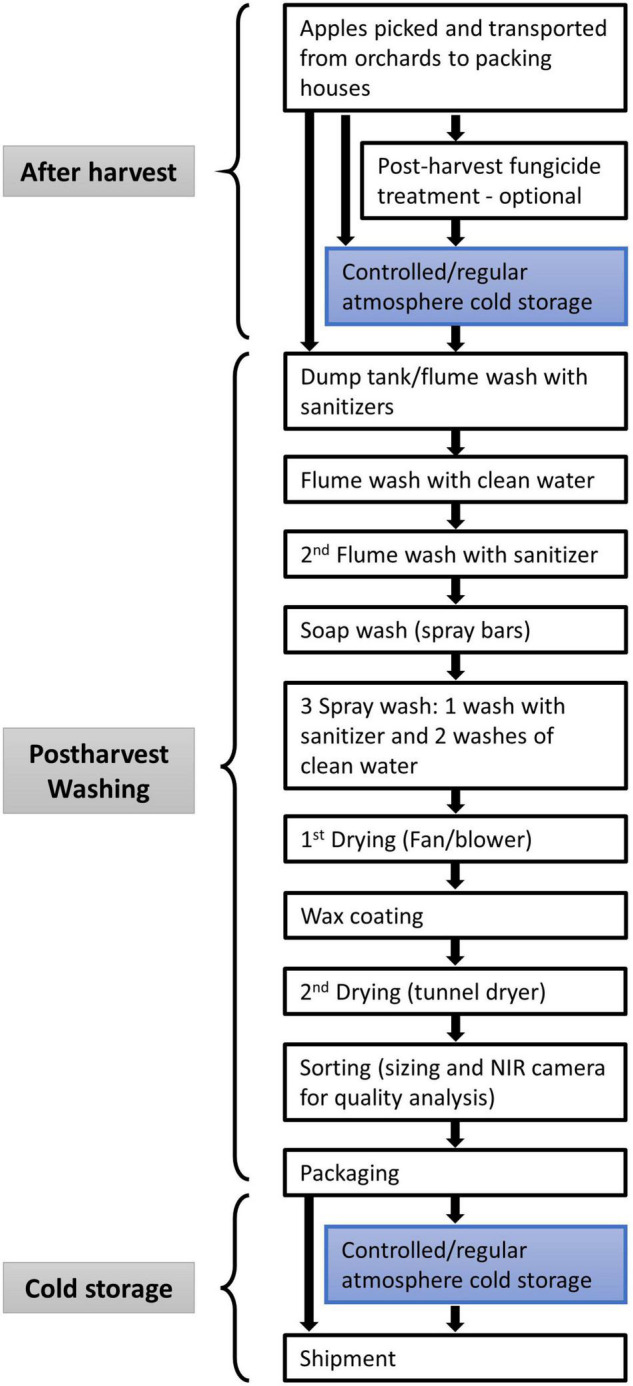
Typical apple packing process in Washington, United States.

After being picked and transported from the orchard to the packing house, fresh apples are either directly washed and packed for shipment or stored in cold storage for future packing (Figure 1). Postharvest fungicide treatment (drenching or fogging) is often applied to fresh apples to avoid mold spoilage for extended storage time. However, the reuse of fungicide solutions in the drenching step may cause cross-contamination with pathogens like *L. monocytogenes*. Additionally, there is no elimination step to remove damaged apples (caused during harvest or transportation) before cold storage. The injured apples might promote the survival, growth, and spread of bacteria and fungus during storage (Ruiz-Llacsahuanga et al., 2021). Thus the preventive controls to reduce the microbial load on apples would be necessary to integrate into the cold storage before washing.

### Postharvest Washing

Postharvest washing is a typical step to reduce contamination for most fresh produce, including processed (i.e., fresh-cut) produce. The aim of most produce washing sanitization on the commercial scale is a 3-log reduction of pathogens on foods and food contact surfaces (Gombas et al., 2017). Various washing steps are used for different apple varieties and fruit quality. Fresh apples in wooden/plastic harvest bins are dumped into flume tanks to remove soil and debris in packing lines. Sanitizers are usually added to the washing water, and the water quality is monitored to avoid cross-contamination. To prevent produce contamination, suitable antimicrobial agents are used, and several runs of freshwater wash are applied (Figure 1). The widely used antimicrobial agents are chlorine, peracetic acid (PAA), aqueous chlorine dioxide (ClO_2_), aqueous ozone (O_3_) and electrolyzed water (EW) (Murray et al., 2017; Sheng et al., 2020). The second flume wash is followed by a soap wash to further remove the dirt and disinfectant remaining on the surfaces. Spray washing intends to use higher pressure to wash off any chemicals or bacteria further.

The industry’s gold-standard sanitizer is sodium hypochlorite for apple postharvest washing. However, the free chlorine reacts with the organic matter in the wash water, reducing efficacy before reaching its target pathogen. U.S. Food and Drug Administration [FDA] (2014) indicated that the commonly used concentrations of hypochlorite (50-200 ppm) maximumly achieve 1 to 2 log reductions on many produce commodities. For example, washing at a 200-ppm concentration of chlorine for 5 min resulted in a 0.6 log reduction for *L. monocytogenes* on whole apples (Beuchat et al., 1998). Recently, potential *Listeria* contamination on fresh apples resulted in voluntary recalls in several states (U.S. Food and Drug Administration [FDA], 2017a, 2019a), suggesting the current washing protocols may not be sufficient for pathogen reduction.

Apples are dried after washing. A typical drying process often includes mild heat with blowing fans. Then, the apples are waxed with an edible coating to improve their appearance and slow down the decay of fresh apples. Before packaging, sizing, and near-infrared (NIR) cameras are used for quality analysis to reject the “bad” apples. After automatic packaging, apples are shipped to the retail markets with minimum storage during the distribution (Pietrysiak et al., 2019; Ruiz-Llacsahuanga et al., 2021).

**FIGURE 2:**
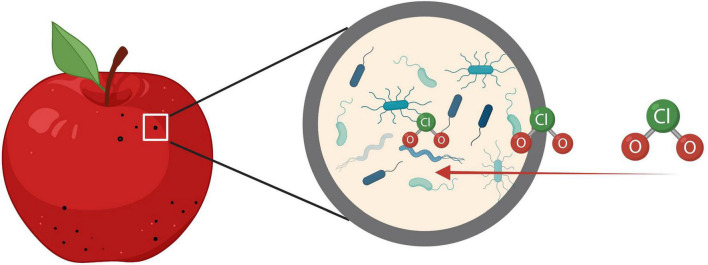
Gaseous chlorine dioxide reaches the bacteria harbored inside the lenticels on apple surfaces.

The challenges on apple decontamination may be due to the naturally irregular shape and microstructures like lenticels (Figure 2), which shield bacteria from the sanitizing interventions (Pietrysiak and Ganjyal, 2018). Gaseous interventions like ClO_2_ have the ability to reach the bacteria harbored inside the lenticels, which could improve sanitation efficacy (Rane et al., 2021). Some lab-scale studies have shown effective log reductions of *Listeria* on apples (Du et al., 2002; Park and Kang, 2017). However, scaling up the methods to fit in the industrial processing line may be challenging due to the line setup and production scale.

### Cold Storage

Two steps of cold storage may take place during the apple packing process (Figure 1). Firstly, the freshly picked apples may be stored in cold storage before they are washed and packaged where cross-contamination may be caused by the damaged apples or happen in environmental conditions (i.e., the facility and the environment). The second storage may happen after packaging before reaching the market. Even if sanitized by washing, the packed apples would contact with bacteria from the environment inside the storage room. The CA cold storage room provides refrigerated temperature, high relative humidity, low oxygen content, circulated air that can facilitate the survival of *L. monocytogenes*.

**FIGURE 3:**
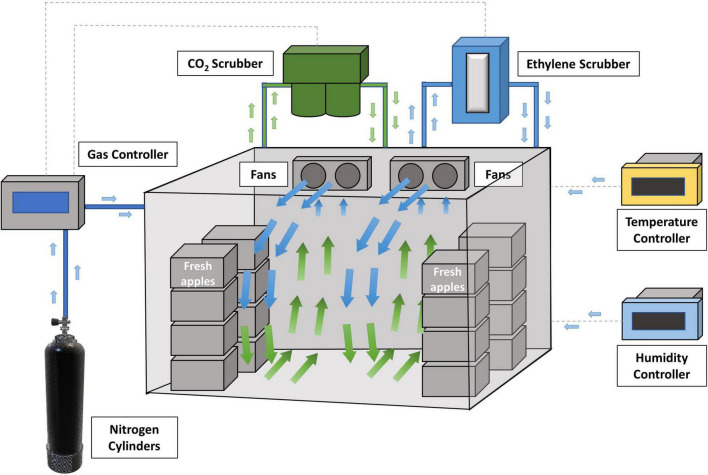
Fresh apples are stored in a controlled atmosphere (CA) cold storage room (adopted from http://www.agroripe.com/controlled-atmosphere-storage/).

Controlled atmosphere (CA) was developed to provide an optimum environment (typically 1-2% O_2_, 1-3% CO_2_, and N_2_) for keeping the freshness of fresh produce and increasing the length of storage by adjustment of normal air composition (78% N_2_, 21% O_2_, 0.03% CO_2_ and other gases). A typical CA cold storage room setup is shown in Figure 3. Optimal CA cold storage reduces the respiration rate and ripening of fresh apples, resulting in maintaining the quality of fruit for up to 11 or 12 months (Farber et al., 2003; Sheng et al., 2018). However, regular atmosphere (RA) or CA cold storage has been designed mainly to extend the shelf-life of fresh apples. *L. monocytogenes* may survive those storage conditions, which becomes a food safety concern. For example, from a previous study (Sheng et al., 2017), *L. monocytogenes* on Fuji apples were decreased by 0.8–1.8 Log_10_ CFU/apple after 3 months of refrigerated atmosphere (RA) storage (1, 4, and 10°C). Sheng et al. (2018) has reported that 30-week of CA cold storage led to 2.5–3.0 Log_10_ CFU/apple reduction of *L. innocua* (surrogate bacteria of *L. monocytogenes*) on Fuji apples. Limited reductions of *Listeria* were observed in both studies. Therefore, RA or CA storage alone is ineffective at controlling *Listeria* on fresh apples, and additional antimicrobial interventions are needed during long-term storage.

## Safety Gaps During Apple Packing Process

Barrera et al. (2012) reported that the actual log reduction of the target pathogen was limited to 1-2 log CFU regardless of the sanitizer or washing time applied, which indicates that the efficacy of postharvest washing is minimal. Various studies showed that the factors that limited efficacy might include pathogen attachment, biofilm formation, and internalization into the plant. Another factor is the dynamics of organic load in the washing flume, which harbors the pathogens from sanitizers and neutralizes part of the antimicrobials, especially relevant in chlorine wash (Shen et al., 2016). The research focus has transitioned from fresh produce decontamination to the prevention of cross-contamination. The concentration of active sanitizer in the wash flume, the quality of water, and organic load have been studied to improve the efficacy of washing, which is challenging in commercial validation (Gombas et al., 2017). Thus, washing itself is insufficient as part of a risk control and prevention approach. Additional or alternative interventions need to be explored and applied to control *L. monocytogenes* in the apple packing process.

Every year, about 4.2 billion pounds of fresh apples are stored in CA cold storage (33-38°F) for up to 12 months, where apples are vulnerable to contamination with *L. monocytogenes* and spoilage microorganisms (Tree Top, 2019). Once contaminated, the apple surface is difficult to decontaminate because of its irregular shape, hydrophobic property, and presence of lenticels (Pietrysiak and Ganjyal, 2018). It is desirable to implement interventions during cold storage as preventive controls. The current existing gas systems in the CA cold storage provide an ideal environment to integrate gaseous interventions to reduce the microbial load on fresh apples. However, there are limited safety interventions implemented during CA cold storage. Furthermore, there is a knowledge gap on integrating current interventions into the CA storage system to ensure safety.

1-Methylcyclopropene (1-MCP) is a widely used inhibitor of ethylene receptors, which delays the ripening of fresh produce products. Commercial application of 1-MCP helps to reduce the ripening process, maintain quality, extend the shelf life of perishable fruits and vegetables (Menniti et al., 2006). 1-MCP can serve as a complementary strategy to CA cold storage to maintain a better quality of the fruit. Lum et al. (2017) reported that 1-MCP in combination with CA cold storage effectively controlled physiologically disordered (senescent scald and internal breakdown) in certain cultivar pear fruits. Mattheis et al. (2017) studied the impacts of 1-MCP and CA storage on the development of bitter pit (physiological disorder) in ‘Honeycrisp’ apples. The results indicated that the use of 1-MCP and/or CA storage can potentially manage the development of bitter pit in ‘Honeycrisp’ apples. Even though 1-MCP fumigation has been combined with CA storage in the apple industry, both methods are applied to preserve food quality. The control of food safety during storage is still missing. Therefore, the integration of an antimicrobial gas to the CA storage can be a potential food safety intervention during the prolonged storage of fresh apples.

## Potential Gaseous Interventions During Cold Storage

In the fresh produce packing industry, different applications of antimicrobial interventions exist throughout the process. Several review papers have summarized various decontamination methods presented in lab-scale studies and used in the fresh produce industry (Deng et al., 2019; Gurtler et al., 2019; Pietrysiak et al., 2019; Marik et al., 2020; Singh et al., 2021; Zhang et al., 2021). Most interventions are still focused on water treatment based on chlorine or alternatives. As water itself can become a pathogen carrier and provide favorable growth conditions for the pathogen, it is well worth outlining the waterless options for the inactivation of *L. monocytogenes* on fresh produce. As one of the waterless methods, gaseous interventions have the advantages to be integrated into the CA cold storage. Potential gaseous interventions include (1) gaseous ClO_2_; (2) gaseous O_3_; and (3) hurdle technology that combines multiple methods. Limited studies have been conducted on gaseous interventions during CA cold storage. Potential interventions that have been applied to fresh apples are summarized in Table 1.

**TABLE 1:** Gaseous food safety interventions for bacteria decontamination on fresh apples.

Interventions	Cold storage highlighted	Food commodity	Pathogen of concern	Conditions	Generation method	Sample mass	Log reduction	Impact on produce quality	References
**Gaseous**									
Chlorine dioxide (ClO_2_)		Fresh apples	*Listeria monocytogenes*	1–8 mg/L, 10–30 min, 21°C, RH = 90–95%	ClO_2_ generator	4 apples	Calyx cavity: 2.8–5.3 log CFU/spotted site; Stem catity: 2.2–5.0 log CFU/spotted site; Pulp surface: 3.5–6.5 log CFU/spotted site.	NM*	Du et al., 2002
		Fresh apples	*Escherichia coli* O157:H7	1.1–18.0mg/L, 10–30 min, 21°C, RH = 90–95%	ClO_2_ generator	4 apples	Calyx cavity: 2.1–6.5 log CFU/site; Stem catity: 1.6–4.1 log CFU/site; Pulp skin: 2.8–7.3 log CFU/site.	NM	Du et al., 2003
		Fresh apples	*Salmonella*	1.4–4.1 mg/L, 6–25 min, 22 ± 1°C, RH = 35–68%	ClO_2_ gas sachets	3 apples	3.21–4.21 log CFU/piece	Subjective evaluation revealed that treatment of apples with 4.1 mg/L ClO_2_ gas for 25 min at 58% relative humidity caused the formation of small brown spots on the skin. The appearance of apples treated with 1.4 and 2.7 mg/L ClO_2_ at 65 to 68% relative humidity was unaffected.	Sy et al., 2005
			Total yeasts and molds				1.09–1.68 log CFU/piece		
		Fresh apples	*Alicyclobacillus acidoterrestis*	0.39–6.55 mg/L peak concentration, 30 min–3 hrs, 22 ± 2°C	ClO_2_ gas sachets	1 apple	2.7–5 log CFU/piece	Treatment with low-release ClO_2_ gas sachets did not affect the visual quality of apples, whereas medium and high-release sachets helped develop small black spots on apple skin.	Lee et al., 2006
		Fresh apples	*Listeria monocytogenes*	20 ppmv, 5–15 min, 22 ± 2°C, RH = 90 ± 2%	ClO_2_ generator	5 × 2 cm pieces	1.47–3.50 log CFU/cm^2^	NM	Park and Kang, 2017
			*Escherichia coli* O157:H7				1.39–4.72 log CFU/cm^2^		
			*Salmonella* Typhimurium				1.25–3.95 log CFU/cm^2^		
Ozone (O_3_)	Cold storage (4–6°C)	Fresh apples	Fungi	1 μl/L for 1 min every 12 hr, 84 days, 4–6°C	O_3_ generator	5 kg	A larger portion of infected apples within the group of ozonated fruits.	Ozone at 1 ppm was unsuccessful in terms of inhibition of fungal disease. However, utilization of ozone slowed down the ripening of apples.	Antos et al., 2018
	Cold storage (sample conditioned at 4°C)	Fresh apples	*Listeria monocytogenes*	23 ppm. 20 min, > 4°C, RH > 85% condensation on the apple surfaces	Forced air ozone reactor	NM	4.26–5.21 log CFU/apple	NM	Murray et al., 2018
	Controlled atmosphere (CA) cold storage (2% O_2_, 1% CO_2_, 0.6°C)	Fresh apples	*Listeria innocua*	50.0–87.0 ppb, 30 weeks, 0.6°C, RH was not actively controlled and expected to be 95% or higher	O_3_ generator	120 apples	2.5–3.0 Log_10_ CFU/apple	Application of gaseous ozone in CA storage did not cause ozone burn or any other unintended side effect on apple fruit quality.	Sheng et al., 2018
			Total bacteria				∼1 Log_10_ CFU/apple		
			Total yeasts & molds				∼0.6 Log_10_ CFU/apple		
		Fresh apples	*Listeria monocytogenes*	77 ppm ± 2 ppm, 15min	O_3_ generator	10 apples	∼3 log CFU/apple	NM	Arévalo Camargo et al., 2019
	Cold storage (12–13°C)		*Lactobacillus*	5 ppm, 40 min, 12–13°C, RH = 55–57%	Forced air ozone reactor	540 kg apples	> 1.5 log CFU/apple		

*NM: not mentioned.

### Gaseous Chlorine Dioxide (ClO_2_)

Gaseous ClO_2_ is a non-thermal and dry antimicrobial process that can be integrated into controlled atmosphere (CA) processes in order to extend shelf life and inactivate foodborne pathogens. There are several advantages of ClO_2_ over traditional chlorine wash (current industry practice), including a higher oxidative capacity (2.5x), enhanced antimicrobial efficacy on porous surfaces, no formation of carcinogenic trichloramine, and reduced corrosion of stainless-steel processing equipment (Wu and Rioux, 2010). Moreover, its higher penetration into the harboring sites of microorganisms or irregular shape of the produce also contributes to the inactivation efficacy (Praeger et al., 2018; Deng et al., 2019).

Gaseous ClO_2_ has been reported to effectively decontaminate foodborne pathogens on various fresh produce (Bridges et al., 2018; Chai et al., 2020; Lacombe et al., 2020; Guan et al., 2021). Gaseous ClO_2_ has been utilized to disinfect *L. monocytogenes* and spoilage microorganisms on fresh apples on a lab scale by Du et al. (2002). This study achieved a reduction of 6.5-log CFU/spotted site *L. monocytogenes* on fresh apples (Du et al., 2002). The survival of *L. monocytogenes* on different spots, including the calyx, stem cavity, and pulp surface of apples, was also studied. *L. monocytogenes* attached to the pulp skin were concluded to be further inactivated by gaseous ClO_2_. Park and Kang (2017) reported that gaseous ClO_2_ could achieve a 3.5 log CFU/cm^2^ reduction of *L. monocytogenes* under 20 parts per million by volume (ppmv) for 15 min. Lee et al. (2006) demonstrated that *Alicyclobacillus acidoterrestis* spores were reduced by 4.5 log CFU/apple by low release gaseous ClO_2_ sachet for 3 h. Treatment with low-release ClO_2_ gas sachets did not affect the visual quality of apples, whereas medium and high release sachets helped with the development of small black spots on apple skin. Sy et al. (2005) studied the decay microorganisms’ survival at 4.1 mg/L gaseous ClO_2_ treatment for 25 min. A 1.68-log CFU/piece reduction of total yeasts and molds was achieved. The treated apples were consistently judged slightly but significantly (α = 0.05) poorer in appearance, color, and overall quality. But the ratings from the sensory panel did not fall below “neither like nor dislike.” These findings agree with the conclusion that gaseous ClO_2_ would effectively inactivate *L. monocytogenes* on fresh apples. However, it is not clear whether the apple quality was sacrificed and what ClO_2_ concentrations may damage the quality.

Decay microorganisms are one of the biggest industry concerns as they cause food waste and economic losses. Gaseous ClO_2_ has been reported to inactivate yeasts and molds, which may help to extend the shelf-life of apples. Application of gaseous ClO_2_ on reducing decay microorganisms of jujube fruit and kiwifruit in cold storage was studied by Park et al. (2019, 2020). An obvious increase in the quality of jujube fruit was observed with a reduction of 1.1-log CFU/g of total bacteria under 50 mg/L gaseous ClO_2_ at 2 ± 1°C (Park et al., 2020). Similarly, Park et al. (2019) indicated that decay incidence and growth of microorganisms were reduced, and the ripening process was retarded under 30 mg/L gaseous ClO_2_ treatment for 30 min. Reductions of 1 log CFU/g, 1.4 log CFU/g, and 0.6 log CFU/g were achieved on total bacteria, total yeasts, and total molds of kiwifruit, respectively. These results demonstrated that gaseous ClO_2_ treatment during cold storage could be a promising decontamination method to reduce microbial load on fruits and maintain the quality.

However, there is a knowledge gap between lab-scale experiments and commercial-scale applications. Predictive models can be a useful tool to bridge the gap. The target reduction of 3-log of pathogens on foods and food contact surfaces and the lab-scale experimental data can be collected to establish the models (Gombas et al., 2017). By modeling the inactivation response of *L. innocua* under different doses, the amount of gaseous ClO_2_ needed in the scale-up study can be calculated via the models. The final step is to integrate gaseous ClO_2_ into the commercial CA cold storage for the decontamination of the *Listeria*-inoculated apples with the predicted conditions. As a result, the fresh apple industry can use the validated models and the engineering setup to apply gaseous ClO_2_ disinfection during their long-term CA storage.

### Gaseous Ozone (O_3_)

Gaseous O_3_ is one of the most powerful oxidizers among food industrial-use sanitizers. The main advantages of O_3_ are the higher efficacy at low concentration over other antimicrobial agents and no residue formation because of decomposition to oxygen. Due to its high reactive and explosive character, O_3_ is unstable and can only be generated right before use. Numerous studies have demonstrated the efficacy of O_3_ inactivation on *L. monocytogenes* (Murray et al., 2018; Sheng et al., 2018).

Several studies integrated gaseous O_3_ into the RA or CA cold storage of fresh apples for bacteria inactivation. Murray et al. (2018) reported that 23 ppm of forced air ozone treatment for 20 min could result in 3.07 log CFU/apple reductions of *L. monocytogenes* on fresh apples taken out of the fridge at 4°C. A commercial-scale ozone treatment on fresh apples was conducted to inactivate *L. innocua* and total bacteria, yeasts, and molds during CA cold storage. *L. innocua* was reduced by 3.0 log CFU/apple under 50-87 ppb ozone treatment at 0.6°C for 30 weeks. Under the same condition, total bacteria and yeasts and molds were reduced approx. 1 and 0.6 log CFU/apple, respectively. Application of gaseous ozone in CA storage did not cause ozone burn or any other unintended side effect on apple fruit quality (Sheng et al., 2018). Another commercial-scale research was conducted by Arévalo Camargo et al. (2019). A forced-air ozone reactor was used during the cold storage of fresh apples to decontaminate *Lactobacillus*, which was selected and validated as the surrogate of *L. monocytogenes* in the same study. Two plastic vented bins containing 540 kg of apples were treated with 5 ppm ozone for 40 min, resulting in more than 1.5 log CFU/apple reductions.

Even though gaseous ozone has been applied under semi-commercial scale CA cold storage, ozone was found unstable since it decomposes fast after generation (Brodowska et al., 2018). In addition, a higher investment in equipment (generator and gas tanks) is required to set up the ozone system. As a strong oxidizer, ozone is more corrosive, particularly on rubber, plastics, and steel (Smilanick, 2003). In the past, gaseous ozone treatments have “burned” overexposed apples during long-term storage (Antos et al., 2018). Therefore, future studies are needed to overcome the difficulties and apply gaseous ozone to control *L. monocytogenes* in CA cold storage.

### Hurdle Technology

Hurdle technology combines different methods to preserve a higher quality of fresh produce for extended shelf-life or to achieve higher efficacy of bacterial decontamination to enhance food safety. Studies on the effectiveness of hurdle technology (gaseous intervention involved) against bacteria on produce are summarized in Table 2. Gaseous ClO_2_ (50 mg/L) and sodium diacetate (200 mg/kg) were combined with CA cold storage (0 ± 1°C) of fresh walnuts to control mold during 135 d of storage. CA cold storage plus ClO_2_ was the optimal treatment and kept the quality of fresh walnuts for 135 d at 0 ± 1°C, with the lowest mold incidence (5%), the highest firmness and contents of fat and melatonin, as well as the maximum peroxidase activity (Ma et al., 2020). Park et al. (2018) reported a hurdle technology of ClO_2_ with UV-C radiation to inactivate *L. monocytogenes* on spinach leaves and tomato surfaces. The combination of UVC and 10 ppmv ClO_2_ were applied on 5 × 2 cm of samples (spinach leaves and tomatoes) for 20 min, which resulted in 4.32 log CFU/g reductions on spinach leaves and undetectable on tomato surfaces after treatment. In this study, the treatments did not significantly (*p* > 0.05) affect the color and texture of samples during storage at 7°C for 7 days. Therefore, the hurdle technology of multiple decontamination methods could potentially reduce *L. monocytogenes* in the apple packing process.

**TABLE 2:** Hurdle technologies (gaseous intervention involved) for bacteria decontamination on produce.

Interventions	Cold storage highlighted	Food commodity	Pathogen of concern	Conditions	Generation method	Sample mass	Log reduction	Impact on produce quality	References
**Hurdle technology**									
Gaseous ozone (O_3_) and hot water		Cantaloupe melon	Mesophilic bacteria	Water (75°C) + air dry (15 min) + O_3_ (10,000 ppm, 30 min, 11°C, RH = 90–95%)	O_3_ generator	6 whole melons	3.8 log CFU/g	No evidence of damage in melons treated with hot water, ozone, or their combination and they maintained initial texture and aroma.	Selma et al., 2008
			Psychrotrophic bacteria				5.1 log CFU/g		
			Molds				2.2 log CFU/g		
			Coliforms				2.3 log CFU/g		
Chlorine dioxide gas (ClO_2_) and aerosolized peracetic acid (PAA)		Spinach leaves	*Escherichia coli* O157:H7	80 ppm PAA + 5/10 ppmv ClO_2_, 5–20 min, 22 ± 2°C, RH = 90 ± 2%	ClO_2_ generator + a commercial ultrasonic nebulizer	5 × 3 cm in size	0.9–5.4 log CFU/g	Combined treatment of ClO_2_ gas (10 ppmv) and aerosolized PAA (80 ppm) did not significantly (p > 0.05) affect the color and texture of samples during 7 days of storage.	Park and Kang, 2015
			*Salmonella* Typhimurium				0.8–5.1 log CFU/g		
			*Listeria monocytogenes*				0.3–4.1 log CFU/g		
		Tomatoes	*Escherichia coli* O157:H7			5 × 2 cm pieces	1.0–5.1 log CFU/g		
			*Salmonella* Typhimurium				0.9–5.2 log CFU/g		
			*Listeria monocytogenes*				0.4–4.5 log CFU/g		
ClO_2_ gas and freezing		Blueberry	Mesophilic aerobic bacteria (MAB)	ClO_2_ gas (4 mg/L, 12 h, 12–14°C) + processing + freezing (-20°C quick, intermediate, slow)	ClO_2_ sachet	16 lugs of blueberries (∼9.1 kg/lug)	2 log CFU/g	ClO_2_ gassing followed by quick freezing effectively meets the current microbiological standards being imposed by buyers of frozen blueberries.	Zhang et al., 2015
			Yeasts and molds				1 log CFU/g		
ClO_2_ gas, ultraviolet-C (UV-C) light, and fumaric acid		Plum	*Escherichia coli* O157:H7	15–30 ppmv ClO_2_ gas, 0.5% fumaric acid, and 10 kJ/m^2^ UV-C, 5–20 min, RH = 80%	ClO_2_ gas generator + UV germicidal lamps	20 ± 0.3 g	4.37–5.48 log CFU/g	The optimal treatment condition does not affect the quality of plum samples.	Kim and Song, 2017
			*Listeria monocytogenes*				5.36–6.26 log CFU/g		
ClO_2_ gas with UV-C radiation		Spinach leaves	*Listeria monocytogenes*	UVC + 10 ppmv ClO_2_ gas, 20 min, 22 ± 1°C, RH = 90 ± 2%	ClO_2_ generator + UV lamp	5 × 2 cm in size	4.32 ± 0.52 log CFU/g	Did not significantly (p > 0.05) affect the color and texture of samples during storage at 7°C for 7 days.	Park et al., 2018
		Tomato surfaces				5 × 2 cm pieces	Not Detectable (ND)		
UV + gaseous O_3_ + hydrogen peroxide		Fresh apples	*Listeria monocytogenes*	UV-C light (54-mJ cm^2^ dose), 6% (v/v) hydrogen peroxide, 2 g/h ozone, 30–120 s, 48°C, RH > 85%	UV-C lamps + ozone-emitting lamps + vaporizimg unit	3 apples	3 log CFU/apple	NM*	Murray et al., 2018
Gaseous ClO_2_ + an edible coating	Cold storage (6°C)	Cantaloupe	*Salmonella*	Gaseous ClO_2_ (5 mg/L, 4.5 h, 6°C, RH = 75%) + NatureSeal edible coating (NS) + cold storage (4°C)	ClO_2_ generator	10 whole cantaloupes	Negative for *Salmonella* after 21 days of storage (detection limit = 2 CFU/g)	For the sensory quality parameters analyzed (color, water loss, and texture), the samples treated with NatureSeal had significantly better quality (p > 0.05) than did the control samples.	Alicea et al., 2018
Gaseous ClO_2_ + cold storage	Cold storage (2°C)	Kiwifruit	Total bacteria	ClO_2_ (30 mg/L, 30 min, RH = 75–80%) + 2 ± 1°C	ClO_2_ generator	270 fruits	1 log CFU/g	Decay incidence and growth of microorganisms reduced, and the ripening process retarded.	Park et al., 2019
			Total yeasts				1.4 log CFU/g		
			Total molds				0.6 log CFU/g		
Gaseous O_3_ + UV-C		Persimmon fruits	Fungi	O_3_ (9.81 mg/m^3^, 1–24) + UV-C (24 cm, 0.5 h)	Activated oxygen generator	6 fruits	99.58–100% killing rate	This non-thermal sterilization could alleviate astringency but hadn’t significant effects on other properties, including color, moisture content, water activity, and protopectin.	Chen et al., 2020
Gaseous ClO_2_ + cold storage	Cold storage (2°C)	Jujube fruit	Total bacteria	10, 30, 50 mg/L, 2 ± 1°C, RH = 80%	ClO_2_ generator	5 kg (35 fruits per sample)	1.1 log CFU/g	An obvious increase in quality.	Park et al., 2020
			Total yeasts and molds				Significantly reduced		
Gaseous ClO_2_ and sodium diacetate (SDA)	Controlled atmosphere (CA) cold storage (2% O_2_ + 25% CO_2_, 0°C)	Fresh walnuts	Mold	CA + 50 mg/L ClO_2_, 0 ± 1°C, 135 d, RH = 70–80%	ClO_2_ powder + water	200 fresh nuts	Mold in the CA + SDA, and CA + ClO_2_ treatments were not detected until day 135	CA + ClO_2_ was the optimal treatment and kept the quality of fresh walnuts for 135 d at 0 ± 1°C, with the lowest mold incidence (5%), the highest firmness, and contents of fat and melatonin, as well as the maximum POD activity.	Ma et al., 2020
				CA + 200 mg/kg SDA, 0 ± 1°C, 135 d, RH = 70–80%	Directly purchased				
Gaseous ClO_2_ + moisture + mild heat		Almond	*Salmonella*	ClO_2_ (20-g precursor dose) + moisture content (7%) + mild heat (40 ± 1.5°C), 1–4 h	ClO_2_ sachet	400 g	2.0 log CFU/g	No visual damages were observed on almonds post-treatment	Rane et al., 2021
			*Enterococcus faecium* NRRL B-2354				1.6 log CFU/g		
Gaseous O_3_ + ultrasonic-assisted aerosolization sanitizer		Lettuce	*Escherichia coli* O157:H7	Gaseous O_3_ (4 and 8 ppm, 3 min) + sodium hypochlorite (SH, 100 and 200 ppm)/acetic acid (AA, 1% and 2%)/lactic acid (LA, 1% and 2%)	Ozone generator + ultrasonic-assisted nebulizer	10 g	0.7 log CFU/g	Quality analysis indicates that LA + 8 ppm ozone and SH + 8 ppm ozone did not negatively affect color, polyphenolic content, weight loss, and sensory properties; however, the levels of two individual phenolic responsible for phenylpropanoid synthesis were significantly increased after treatment with 2% LA + 8 ppm ozone.	Wang et al., 2021
			*Salmonella* Typhimurium				0.75–1.28 log CFU/g		
			*Listeria monocytogenes*				0.58 log CFU/g		
Gaseous ClO_2_ + 1-methylciclopropene (1-MCP)	Cold storage (4°C)	Sweet cherry	Fungi	ClO_2_ (30 μL/L) + 1-MCP (1 μL/L), 24 h, 4°C	Release from solid ClO_2_ + release from 1-MCP powder formulation	4 kg	11.7% decay incidence (more than 38.9% decrease)	Better improve the postharvest quality of sweet cherry fruit.	Zhao et al., 2021

*NM: not mentioned.

## Federal Regulations

### Food Safety Modernization Act

Ready-To-Eat (RTE) foods represent foods that are eaten without any further processing to reduce the microbiological hazards. RTE foods that have intrinsic characteristics (such as pH and water activity) can be natural or processed to prevent the growth of *L. monocytogenes*. As one of the RTE foods, apples are naturally preventing the growth of *L. monocytogenes* since the pH is less than 4.4 (U.S. Food and Drug Administration [FDA], 2017b). U.S. Food and Drug Administration [FDA] (2008) considers the adulteration of *L. monocytogenes* on a food product that contains more than 100 colony forming units (CFU) per gram of food when an RTE product does not support the growth of *L. monocytogenes* (Smith et al., 2018). Additionally, there is a zero-tolerance of *L. monocytogenes* if the RTE food supports *L. monocytogenes* growth.

Since fresh apples are considered both produce and Raw Agricultural Commodity (RAC), fresh apple packing house falls under the FDA Food Safety Modernization Act (FSMA) Final Produce Safety Rule (PSR). RAC means any food in its raw or natural state. All fruits, including fresh apples that are washed, colored, or treated in their unpeeled natural state prior to marketing, is considered as RAC (U.S. Food and Drug Administration [FDA], 2008). When a particular RAC is made into processed food, for example, irradiated papayas, the PSR applies only when the fresh papaya is a RAC before irradiation. When signed into law in 2011, FSMA highlighted the importance of preventive controls to reduce the incidence of food contamination that can lead to foodborne illness and outbreaks (U.S. Food and Drug Administration [FDA], 2011).

As a part of FSMA, PSR demonstrates the science-based requirements throughout the safe growing, harvesting, packing, and holding of produce grown for human consumption. The final rule went into effect in 2016. The rule requires the covered farms to take appropriate actions to minimize the risk of severe health consequences or death from the exposure to covered produce. It also requires the necessary prevention of the introduction of known or reasonably foreseeable hazards into the produce and providing suitable controls that save the produce from being adulterated. A Food Safety Plan (FSP), required by the FSMA Preventive Controls for Human Food (PCHF), documents a systematic approach to identify the food safety hazards that must be controlled to prevent or minimize the risk of foodborne illness. It is challenging for the produce industry to comply with the requirements since there is no “kill” step to eliminate pathogens. Therefore, identification of safety hazards and implementation of sufficient cleaning and sanitation preventive controls (i.e., safety interventions) draws great attention (U.S. Food and Drug Administration [FDA], 2016).

### Residue Limits

The major ClO_2_ disinfectant by-products (DBPs) of concern are chlorite (ClO^2–^) and chlorate (ClO_3_^–^) ions, with no direct formation of organohalogen DBPs. Unlike the other disinfectants, the major ClO_2_ DBPs are derived from the decomposition of the disinfectant as opposed to reaction with precursors (World Health Organization [WHO], 2000). The maximum contaminant level (MCL) of chlorite in drinking water, is 1.0 mg/L (U.S. Environmental Protection Agency [EPA], 2011).

In a recent study, the European Food Safety Authority investigated the presence of residues of chlorate in food and drinking water. The data showed that the chlorate exposure exceeded the tolerable daily intake has negatively impacted the iodine uptake, especially among infants and young children. In order to reduce the chlorate levels, the European Commission published new regulations of maximum residue levels for chlorate and perchlorate in foods in the summer of 2020. The maximum allowed level of chlorate on apples is 0.05 mg/kg. Perchlorate mainly affects fruits and vegetables. The maximum allowed level is 0.05 mg/kg. These new regulations highlighted the importance of monitoring chlorate and perchlorate residues after chlorine-based sanitation and reducing the concentration of disinfectant used in or on food products (European Commission [EC], 2020a,b).

Environmental Protection Agency Final Rule (EPA–HQ–OPP–2017–0063) was effective on December 26, 2018. 40 CFR Part 180 (Federal Register Number: 2018-27908) stated that “Residues of chlorate in or on tomato and cantaloupe are exempt from the requirement of a tolerance when resulting from the application of gaseous chlorine dioxide as a fungicide, bactericide, and antimicrobial pesticide,” which allows for the expanded use of gaseous ClO_2_ on fresh produce. Other food commodities might be exempted with more data and scientific support in the future (U.S. Environmental Protection Agency [EPA], 2018).

### Organic Production and Handling

The U.S. Department of Agriculture (USDA) has established a National Organic Program (NOP) rule to enforce organic production and handling requirements. The NOP provides the guidance of “The Use of Chlorine Materials in Organic Production and Handling” to state that approved chlorine materials may be utilized in direct contact with organic production according to label directions. Allowed chlorine materials in organic production are calcium hypochlorite, ClO_2_, and sodium hypochlorite. Chlorine use must be immediately followed by a rinse sufficient to reduce chlorine levels on the product to potable water levels at the maximum residual disinfectant level of 4 mg/L for chlorine (as Cl_2_) and 0.8 mg/L for ClO_2_, which is currently established by the Environmental Protection Agency (EPA) at 40 CFR § § 141.2, 141.65 (U.S. Department of Agriculture [USDA], 2011; U.S. Environmental Protection Agency [EPA], 2011).

However, ClO_2_ is currently allowed for use in liquid solution in crop production as a preharvest algicide, disinfectant, and sanitizer, including in irrigation system cleaning systems (7 CFR § 205.601(a)(2)(ii)); in organic livestock production for use in disinfecting and sanitizing facilities and equipment (7 CFR § 205.603 (a)(7)(ii)); and in organic handling for disinfecting and sanitizing food contact surfaces (7 CFR § 205.605(b)). For these uses, residual chlorine levels in the water cannot exceed the maximum residual disinfectant limit under the Safe Water Drinking Act. A petition was submitted to NOP to extend the use of ClO_2_ in gaseous form for the antimicrobial treatment of products labeled “organic” or “made with organic [specified ingredients or food group(s)]” in 2015. This petition was transmitted to National Organic Standards Board (NOSB) in U.S. Department of Agriculture [USDA] (2018).

The current regulation status indicates that ClO_2_ in its liquid form under the guidance has been considered organic production, whereas in its gaseous form has not yet been approved. More studies on gaseous ClO_2_ and the demand for higher efficacy sanitation from the food industry may facilitate the approval of the petition.

O_3_, as one of the approved chemicals for use in organic postharvest systems, is considered GRAS (Generally Recognized as Safe) for produce and equipment disinfection. Exposure limits for worker safety apply (U.S. Department of Agriculture [USDA], 2011). The Occupational Safety and Health Administration (OSHA) regulates employee exposure to O_3_ gas through its Air Contaminants Standard, 29 CFR 1910.1000. The permissible exposure limit (PEL) is an 8-h, time-weighted average value of 0.1 part of O_3_ per million parts of air (ppm) (Occupational Safety and Health Administration [OSHA], 1994).

## Conclusion

During the postharvest packing process, a heat-based lethal treatment cannot be applied to fresh produce like apples. As a persistent and pathogenic microorganism, *L. monocytogenes* has caused high risk contamination in fresh produce packing facilities in previous outbreaks. The current packing process mostly relies on postharvest washing has been reported to be insufficient for produce decontamination. Additionally, the long-term CA cold storage of fresh apples provides optimal conditions for *Listeria* growth and persistence. Therefore, potential safety interventions to inactivate *L. monocytogenes* during cold storage are in great need.

Waterless safety intervention and hurdle technology can be future directions to help improve the apple decontamination efficiency. Water treatment has brought numerous problems like reacting with organic loads, cross-contamination, and abundant water usage. In comparison, waterless treatments may avoid the problems and increase the effectiveness of the antimicrobials. However, waterless interventions might have different problems like residue allowance, workers’ safety concerns, etc. The related regulations are still undergoing review. Both water and waterless treatments lack methodological standards, making it hard to compare data from different studies.

The integration of gaseous ClO_2_ into industrial CA cold storage offers critical food safety benefits for fresh apples by reducing the risk of pathogen contamination during storage. But it is necessary to ensure that ClO_2_ does not induce any lenticel breakdown nor bitter pit symptoms. Since the lenticels can harbor bacteria, thus protecting them from antimicrobial interventions, the gas interventions should inactivate pathogens without causing tissue damage. The overall fruit quality after gaseous ClO_2_ treatment needs to be inspected for any negative impact. Dry media generation of ClO_2_ avoids overdosing through control-released technology. However, there is a lack of knowledge with regards to the optimum initial dose to prevent any damage to the fruits. In addition, recent studies have concluded that a slow controlled release of ClO_2_ using dry precursors resulted in undetectable amounts of chlorate and chlorite residues (Smith and Scapanski, 2020). The lack of chemical residue is very important in order to maintain industry standards. The new commercial-scale integration system could augment GMP safety plans by reinforcing critical control points that rely heavily on postharvest washing. Apple growers and processors can use this in a storage decontamination step as part of the hurdle technology safety plans, helping to secure food safety and reduce food waste and economic losses. Therefore, gaseous ClO_2_ integrated into CA cold storage of fresh apples can potentially control *L. monocytogenes*.

Significant research is still greatly needed to develop effective methods to reduce microbial loads on fresh apples. Critical aspects, including surface characteristics of apples, commercial-scale validation of the intervention, intervention implementation/integration to the current existing apple packing process, and the impact of the interventions on final apple quality, should be taken into consideration.

## Author Contributions

JG contributed to the conceptualization and design, reviewed the literature, prepared the figures and tables, and wrote the original draft of the manuscript. AL contributed to the conceptualization and design, and wrote, reviewed, and edited the manuscript. BR and SS wrote, reviewed, and edited the manuscript. JT supervised the data and wrote, reviewed, and edited the manuscript. VW contributed to the conceptualization, supervised the data, carried out the project administration, funding acquisition, and resources, and wrote, reviewed, and edited the manuscript. All authors contributed to the article and approved the submitted version.

## Conflict of Interest

The authors declare that the research was conducted in the absence of any commercial or financial relationships that could be construed as a potential conflict of interest.

## Publisher’s Note

All claims expressed in this article are solely those of the authors and do not necessarily represent those of their affiliated organizations, or those of the publisher, the editors and the reviewers. Any product that may be evaluated in this article, or claim that may be made by its manufacturer, is not guaranteed or endorsed by the publisher.
